# Diagnosis of Alzheimer’s Disease with Ensemble Learning Classifier and 3D Convolutional Neural Network

**DOI:** 10.3390/s21227634

**Published:** 2021-11-17

**Authors:** Peng Zhang, Shukuan Lin, Jianzhong Qiao, Yue Tu

**Affiliations:** Department of Computer Science and Engineering, Northeastern University, Shenyang 110819, China; zhangpeng.coder@gmail.com (P.Z.); qiaojianzhong@cse.neu.edu.cn (J.Q.); 1810615@stu.neu.edu.cn (Y.T.)

**Keywords:** Alzheimer’s disease, convolutional neural network, ensemble learning, data denoising

## Abstract

Alzheimer’s disease (AD), the most common type of dementia, is a progressive disease beginning with mild memory loss, possibly leading to loss of the ability to carry on a conversation and respond to environments. It can seriously affect a person’s ability to carry out daily activities. Therefore, early diagnosis of AD is conducive to better treatment and avoiding further deterioration of the disease. Magnetic resonance imaging (MRI) has become the main tool for humans to study brain tissues. It can clearly reflect the internal structure of a brain and plays an important role in the diagnosis of Alzheimer’s disease. MRI data is widely used for disease diagnosis. In this paper, based on MRI data, a method combining a 3D convolutional neural network and ensemble learning is proposed to improve the diagnosis accuracy. Then, a data denoising module is proposed to reduce boundary noise. The experimental results on ADNI dataset demonstrate that the model proposed in this paper improves the training speed of the neural network and achieves 95.2% accuracy in AD vs. NC (normal control) task and 77.8% accuracy in sMCI (stable mild cognitive impairment) vs. pMCI (progressive mild cognitive impairment) task in the diagnosis of Alzheimer’s disease.

## 1. Introduction

Alzheimer’s disease (AD) is a progressive neurodegenerative disease with insidious onset. Clinically, it is characterized by general dementia such as memory impairment, aphasia, impairment of visuospatial skills, and executive dysfunction. A global Alzheimer’s disease report from the International Association for Alzheimer’s disease pointed out that the number of AD patients in the world has increased dramatically. It may increase from 47 million in 2015 to 130 million in 2050, and the cost of AD treatment will also increase sharply, from 800 billion in 2015 to 2 trillion in 2030. This may bring a serious economic burden to the world. The elderly have a high incidence of Alzheimer’s disease, and the aging of the population has become a serious social problem. Therefore, the elderly population with Alzheimer’s disease will increase. Computer-aided diagnosis (CAD) of AD can help to detect the disease as early as possible and inhibit its progression, which has important significance in research and practical applications.

Mild cognitive impairment (MCI) is a prodromal state of dementia, which is the most important and suitable stage for AD preventive treatment. MCI is divided into progressive mild cognitive impairment (pMCI) and stable mild cognitive impairment (sMCI), where pMCI will eventually deteriorate into AD within 36 months, and sMCI is in a relatively stable condition with no lesions.

Magnetic resonance imaging (MRI) is a main medical neuroimaging method, which is an important basis for the diagnosis of Alzheimer’s disease. Compared with computed tomography (CT), the organization structure of MRI is clearer. Brain morphometric pattern analysis using MRI data are proven to be effective in identifying anatomical differences between populations of AD patients and normal controls (NC). In recent years, deep learning has achieved significant results in the field of computer vision, especially in the use of convolutional neural networks (CNNs) [1] to extract image features, which provides new ideas for AD diagnosis [2]. Many researchers have built 3D CNN models for AD diagnosis based on 3D MRI data, which contains more information compared with 2D data. However, 3D CNNs need a lot of training time. Some existing methods, for example, the voxel-based ones [3,4], patch-based ones [5,6] and ROI-based ones [7,8], tried to use part of an image for feature extraction [9,10,11], which require good priori knowledge to know which part affects the diagnosis of Alzheimer’s disease. Because the etiology and pathology of Alzheimer’s disease are not clear, these methods may reduce diagnosis accuracy. Some methods used whole images for feature extraction [12], which greatly increases calculation. Besides, most of the existing methods employed a single classifier for AD diagnosis, which limits the further improvement of diagnosis performance. Moreover, a limited number of MRI images were not fully used for AD diagnosis in currents models. Especially, there are few studies on hard sample processing to improve the diagnosis accuracy of Alzheimer’s disease. Therefore, although the existing methods have achieved good diagnostic results, there are still some problems that remain to be solved. The diagnosis of Alzheimer’s disease has the following challenges:

**Challenge 1:** Although 3D MRI data contain more information than 2D data, the 3D MRI image-based 3D CNN models need more training time. Therefore, how to reduce the calculation in 3D CNN modeling without losing much useful information is a challenge for AD diagnosis. By observing 3D MRI images, we found that there are many noise regions near their edges. These noise regions contain a large number of useless boundary voxels. The introduction of a large amount of noise not only slows down the training speed, but also reduces diagnostic accuracy. Therefore, how to process the noise regions to increase training speed while improving diagnostic accuracy is a problem to be solved when we build our 3D CNN diagnosis model.

**Challenge 2:** For 3D MRI images, a single classifier limits the performance improvement of AD diagnosis. Meanwhile, inefficient utilization of a limited number of MRI images will lead to overfitting in the 3D CNN diagnosis models while hard samples usually reduce the accuracies of the models. Therefore, how to break through the limitation of using a single classifier and process a hard sample while fully utilizing the limited MRI images to improve diagnostic performance is another challenge. In our study, for this challenge, how to construct the corresponding 3D CNN network architecture and learn hard samples is a problem to be solved.

To tackle the above problems, we propose a novel diagnostic model with a data denoising module and diagnosis network module. The major contributions of this paper can be summarized as follows.

1 To process the noise regions to increase training speed while improving diagnostic accuracy, we propose a data denoising method that reduces the noises at the boundaries of the 3D MRI images through the designed clipping algorithm. By analyzing the differences between the images of different label groups, the difference image is obtained, and the image boundary is cropped according to the normalized mask threshold to reduce noises. The data denoising method can remove about 47% of useless information from the data for AD diagnosis, which not only speeds up the training process but also improves the accuracy of diagnosis.
2 Considering the limitation of a single classifier, the problem of hard sample learning and the issue of insufficient sample utilization, we propose a diagnosis network with multiple classifiers and serialization learning, where samples are repeatedly used in various classifiers with different weights according to their prediction errors in the previous classifier. Hard samples are assigned larger weights due to their larger prediction errors so that we can focus on them. The diagnosis network is trained based on the ensemble learning method with an improved loss function and adaptive fusion.
3 The experimental results demonstrate that our proposed method outperforms the state-of-the-art methods regarding both accuracy and efficiency. Specifically, our proposed method has achieved a high accuracy 95.2% (AD vs. NC) and 77.8% (sMCI vs. pMCI).

The rest of this paper is organized as follows. We briefly introduce the most relevant studies in Section 2. In Section 3, we describe the data used in this study and present our method in detail. In Section 4, we show experimental results and performance evaluation. Finally, the conclusions are given in Section 5.

## 2. Related Work

A key component of an MRI-based AD diagnosis system is to determine how to extract features from MRI images. Generally speaking, the feature extraction of existing AD/MCI diagnosis can be roughly divided into four categories, including voxel-based feature extraction, patch-based feature extraction, region-of-interest (ROI) based feature extraction, and whole-image-based feature extraction.

More specifically, the voxel-based features measure the local tissues associated with AD [13]. Baron et al. [3] proposed a feature extraction method based on voxels and utilized the statistical features of voxels for diagnosis. Li et al. [4] used the voxel information of the cerebral cortex to extract features and input them into a support vector machine (SVM) [14] for diagnosis. Compared with a relatively small number of images (such as tens or hundreds) used for model training, the feature dimension of voxels is very high (such as millions). This kind of method usually faces the challenge of overfitting.

The patch-based methods use all or part of patches as inputs for diagnosis [15]. Zhang et al. [5] used the regression forest algorithm to predict the locations of AD lesions, then cropped MRI images according to these locations, and finally input them to SVM for diagnosis. Liu et al. [6] used positron emission computed tomography (PET) and MRI images for multi-modal diagnosis. Besides, they divided each MRI and PET image into patches according to corresponding positions and input to a cascade network. Liu et al. [16] identified discriminative anatomical landmarks from each MRI image via a data-driven strategy and extracted multiple patches around these landmarks, which are input into multiple classification networks for multi-instance diagnosis learning. Pan et al. [17] used generative adversarial networks (GAN) to generate missing PET data, and then divided an image into multiple patches to input to a multi-instance neural network for learning. Suk et al. [18] used multi-modal data and divided them into multiple patches to input into the deep network for fusion diagnosis. Lian et al. [19] pre-determined the location of each lesion to generate the corresponding patch, and input them to CNN. Ortiz et al. [20] split gray matter images from each brain area into 3D patches according to the regions defined by the automated anatomical labeling atlas and these patches are used to train different deep belief networks. Although these patch-based methods can reduce the number of network parameters, the information between patches is relatively independent, which affects the diagnostic effectiveness.

The ROI-based methods mainly extract features from pre-segmented brain regions and construct classifiers [21]. Zeng et al. [7] used the gray matter regions in a brain to extract features and proposed an overall framework for optimizing SVM parameters. Wang et al. [8] used brain extraction tools to select the slices containing the hippocampus and input them into a convolutional neural network for diagnosis. Cui et al. [9] used gray matter in a brain to extract features and proposed a classification framework combining convolutional neural networks and recurrent neural networks for longitudinal analysis of structural MRI in AD diagnosis. Ju et al. [10] used functional MRI to divide a brain into 90 different regions for AD diagnosis. Cui et al. [11] diagnosed the disease by analyzing the characteristics of the shape and volume of biomarkers such as the hippocampus. These ROI-based methods can achieve good diagnostic results if the regions are selected correctly. However, region selection is based on prior knowledge, so all possible pathological locations in an entire brain may not be covered.

In the whole-image-based methods, an image is input to a network as a whole [22]. Liu et al. [23] cut 3D MRI data into 2D slices in three directions and input them into CNN and recurrent neural network for fusion diagnosis. Wang et al. [12] input 3D MRI into DenseNet [24] for feature extraction for the first time, and then applied integrated learning for diagnosis. In this paper, five diagnostic subnetworks are designed and a new integrated diagnosis method is proposed. Islam et al. [25] used deep convolutional neural networks to train on a small dataset, and proposed an effective method for training deep learning models with unbalanced data. Maqsood et al. [26] utilized transfer learning to classify images by fine-tuning a pre-trained convolutional network, AlexNet. Karasawa et al. [27] designed a 3D CNN architecture for diagnosing AD and achieved good performance. Basaia et al. [28] augmented training data by deforming, cropping, rotating, flipping and scaling MRI data at different angles, and then input them into a convolutional neural network for diagnosis. Although this kind of method uses all the information of an image, there are some boundary noises, which make calculation expensive. There are also some methods that use other personal information besides images, such as age, gender, and checklist scores. For instance, Duc et al. [29] used a 3D convolutional network to jointly predict patients’ diagnosis categories and their mental status scores. Feng et al. [30] designed a new deep learning framework. Specifically, the framework makes use of the advantages of 3D CNN and the full stacked bidirectional long short term memory network. Fsbi LSTM is used to extract the hidden spatial information in deep feature map to further improve its performance. Although this method makes use of all the information of the image, because the boundary of MRI image has useless information, this calculation cost is greater and unnecessary for the network using 3D MRI.

The existing methods have the following problems: (1) there are a large number of voxels in a 3D MRI image with a large amount of useless boundary noise information, which not only affects the efficiency of 3D CNN modelling but also the accuracy of AD diagnosis. In current works, the useless boundary noise information did not be processed effectively. (2) Most of the existing methods used a single classifier for AD diagnosis, which limits the improvement of diagnosis performance. (3) In existing methods, a limited number of 3D MRI images were not sufficiently utilized for network training, while inputting 3D MRI samples into a network directly did not consider their different importance for diagnosis results. (4) Few existing methods focused on hard sample learning, leading to suboptimal diagnosis results. To tackle the above issues, we first propose a novel denoising method for 3D MRI data, which removes useless noise information. Then, we propose a diagnosis network, which contains multiple 3D CNNs with the corresponding improved loss functions as classifiers and the limited 3D MRI samples are repeatedly utilized in various classifiers with different weights. Especially, by incorporating the idea of AdaBoost [31], the weights of samples can be learned according to their prediction errors in the previous classifier, and hard samples are assigned larger weights due to their larger prediction errors so that we can improve diagnosis performance by focusing on hard sample learning.

## 3. Our Diagnosis Model

### 3.1. Model Architecture

**Figure 1:**
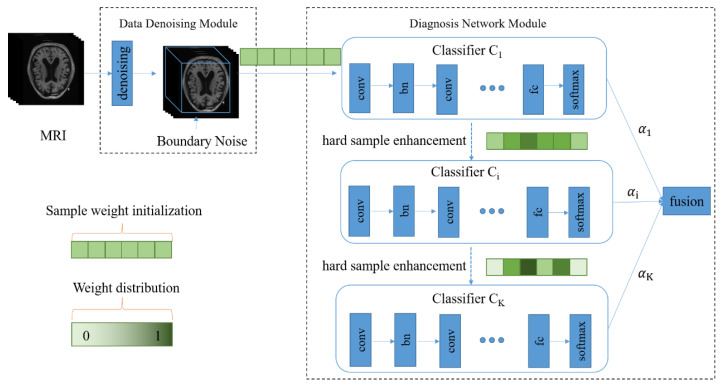
The architecture of the proposed diagnosis model for Alzheimer’s disease. There are two main components, including the data denoising module and diagnosis network module.

As shown in Figure 1, our diagnosis model consists of two major parts, namely, the data denoising module and the diagnosis network module. In Figure 1, the green part represents the weights of the samples, and the color depth represents the weight size. After 3D MRI samples are cropped by the data denoising module, they are input into the diagnosis network containing multiple classifiers. In the training process, the weight of each sample is continually adjusted according to the diagnosis error of the corresponding classifier, and each sample is input to the next classifier together with its updated weight so that hard samples can be assigned high weights in the next learning. Meanwhile, the fusion weight of each classifier is adaptively learned so that the diagnosis results of all classifiers can be adaptively fused in the testing process.

More details about the data denoising module are presented in Section 3.2. In Section 3.3 and Section 3.4, we introduce the diagnosis network including the adaptive fusion and the hard sample learning.

### 3.2. Data Denoising Module

**Figure 2:**
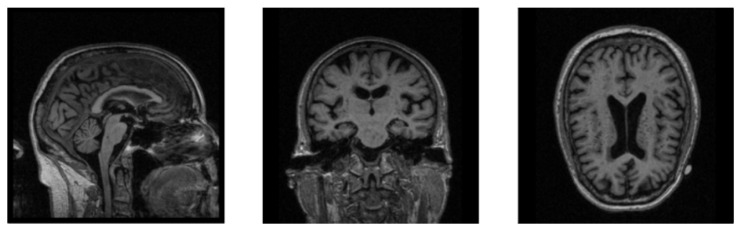
The sectional views of a 3D MRI image along three directions. The three views from left to right are sagittal, coronal, and axial.

The MRI images are mostly 3D data, which contain more information than 2D data while requiring a larger amount of calculation during training, which makes neural networks less efficient when training 3D MRI data. In Figure 2, we show the sectional views of a 3D MRI image along three directions (sagittal plane, coronal plane, and axial plane). We can clearly see that there are many black noise regions near the margins of each image. These regions contain a lot of useless boundary voxels, which have no impact on brain tissues and need to be processed prior to training the neural network. The introduction of a large amount of noise not only slows down the process of model training, but also reduces the accuracy of diagnosis. The motivation of the data denoising module is to provide more valuable data for AD diagnosis as the inputs of our neural network, so as to improve the diagnostic accuracy of our network model. Based on this, a data denoising method is proposed, which removes the marginal noise regions in raw data to accelerate network training and improve diagnostic accuracy.

**Figure 3:**
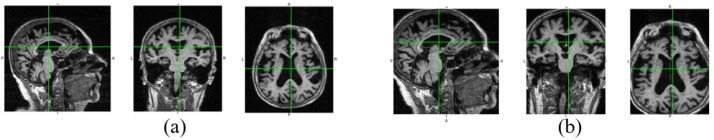
Raw image and the denoised image. The three views of the raw image are shown in (**a**), and (**b**) is the corresponding denoised views.

In Figure 3, we show the data changes before and after data denoising. Through data denoising, the noise regions in the raw 3D image are cut out, and the size of the data input into the neural network can be reduced from 192 × 192 × 160 to 160 × 160 × 120. Because the noise data is reduced, the neural network model can better extract useful features for diagnosis. In addition, data denoising helps to reduce calculation by decreasing parameters of the fully connected layer in our diagnosis network.

In order to better understand the method proposed in this paper, the related concepts are defined as follows.

**Definition 1.** ***(x-Sample image):*** *Given a dataset M of 3D MRI images where there are a total of four types of label values AD, NC, sMCI and pMCI for all sample images and the label value set L = {AD, NC, sMCI, pMCI}, the sample image with the label x ∈ L in M is called x-sample image, denoted by $s_{}^{x}$.*

**Definition 2.** ***(Image group):*** *Given the dataset M, the set $G^{x}\;=\;\left\{s_{1}^{x},s_{2}^{x},\cdot \cdot \cdot ,s_{m}^{x}\right\}\subseteq M$, where $s_{i}^{x}(1\;\leq \;i\;\leq \;m)$ is an x-sample image and m is the number of x-sample images in M, is called x-image group.*

**Definition 3.** ***(Mean image):*** *Given the x-image group $G^{x}\;=\;\left\{s_{1}^{x},s_{2}^{x},\cdot \cdot \cdot ,s_{m}^{x}\right\}\subseteq M$, the mean image $\bar{G^{x}}$ of $G^{x}$ is defined as the image with the average voxel level of $G^{x}$. Each voxel value $\bar{G^{x}}\left(a,b,c\right)$ is calculated as follows:*

(1) $$\bar{G^{x}}\left(a,b,c\right)\;=\;\frac{1}{m}\sum_{i=1}^{m}s_{i}^{x}\left(a,b,c\right),$$

*where $s_{i}^{x}\left(a,b,c\right)$ is the voxel value of the 3D sample image $s_{i}^{x}$.*

**Definition 4.** ***(Difference image):*** *Given the mean images $\bar{G^{x_{1}}}$ and $\bar{G^{x_{2}}}$, the difference image of $\bar{G^{x_{1}}}$ and $\bar{G^{x_{2}}}$ is defined as the image with their voxel difference at each position, denoted by $D_{}^{x_{1}-x_{2}}$. Each voxel value $D_{}^{x_{1}-x_{2}}\left(a,b,c\right)$ is calculated as follows.*

(2) $$D_{}^{x_{1}-x_{2}}\left(a,b,c\right)\;=\;\bar{G_{}^{x_{1}}}\left(a,b,c\right)-\bar{G_{}^{x_{2}}}\left(a,b,c\right).$$

We use the AD-image group $G^{\mathrm{AD}}$ and the NC-image group $G^{\mathrm{NC}}$ to explain our data denoising process. First, we obtain their mean images $\bar{G^{\mathrm{AD}}}$ and $\bar{G^{\mathrm{NC}}}$ according to Equation (1). Then the difference image $D_{}^{\mathrm{AD}-\mathrm{NC}}$ is calculated according to Equation (2). By normalization, we can get the difference image ${\tilde{D}}_{}^{\mathrm{AD}-\mathrm{NC}}$ with the voxel values between 0 and 1. Next we transform ${\tilde{D}}_{}^{\mathrm{AD}-\mathrm{NC}}$ into its corresponding mask according to a given threshold α. Finally, each AD-sample image and each NC-sample image are cropped according to the mask to achieve noise reduction. In this way, the lesion areas corresponding to large differences in an image will be preserved, and the useless voxels can be cut off. In Figure 3, we show the views of a raw image at three dimensions and their corresponding denoised views. It can be found that the useless information near the boundaries is reduced, but the main information is not lost. The implementation details of data denoising are shown in Algorithm 1.
**Algorithm 1:** Data denoising process**Input:** AD-Image group $G^{\mathrm{AD}}\;=\;\left\{s_{1}^{\mathrm{AD}},s_{2}^{\mathrm{AD}},\cdot \cdot \cdot ,s_{m}^{\mathrm{AD}}\right\}$, NC-Image group $G^{\mathrm{NC}}\;=\;\left\{s_{1}^{\mathrm{NC}},s_{2}^{\mathrm{NC}},\cdot \cdot \cdot ,s_{n}^{\mathrm{NC}}\right\}$, the threshold α **Output:** The set *R* of the positions where the voxel values will be preserved in denoising
1:Calculate the mean images $\bar{G^{\mathrm{AD}}}$ and $\bar{G^{\mathrm{NC}}}$2:Calculate the difference image $D_{}^{\mathrm{AD}-\mathrm{NC}}$ of $\bar{G^{\mathrm{AD}}}$ and $\bar{G^{\mathrm{NC}}}$3:Find the maximal value *max* in $D_{}^{\mathrm{AD}-\mathrm{NC}}$4:Each value of $D_{}^{\mathrm{AD}-\mathrm{NC}}$ is divided by *max* to normalize it and obtain ${\tilde{D}}_{}^{\mathrm{AD}-\mathrm{NC}}$5:**for** each position *p* in ${\tilde{D}}_{}^{\mathrm{AD}-\mathrm{NC}}$ **do**6:   **if** $value_{p}$ *>* α **then**
7:      
$mask_{p}\;=\;1$
8:   **else**9:      
$mask_{p}\;=\;0$
10:   **end if**11:**end for**12:Add the coordinates with the value 1 to the set *R*13:**return***R*

### 3.3. Diagnosis Network Module

As we know, medical images available for training are less adequate than ordinary images, so it is more necessary to make full use of the information of each sample in the training process. Especially for 3D medical images, each sample has a lot of voxel points and contains a lot of information. Therefore, the training process should focus on learning misclassified samples. On the one hand, we utilize multiple classifiers to repeatedly train limited MRI images so that they can be fully exploited. On the other hand, we continually adjust the weight of each sample in each classifier according to its prediction error in the previous classifier to punish misclassified samples and improve the accuracy of diagnosis.

#### 3.3.1. The Structure of Diagnosis Network

The data used in this paper are 3D images. Therefore, we build a 3D convolutional diagnosis network so that the correlation between different dimensions is not lost, as shown in Figure 1. The diagnosis network incorporates the idea of ensemble learning and utilizes multiple CNNs as classifiers to achieve adaptive fusion. In order to reduce network parameters, inspired by VGG [32], we design 3 × 3 × 3 small convolution kernels to extract image features in each classifier. We use ReLU [33] as the activation function. ReLU activation function is widely used in DNNs (Deep Neural Networks), especially in CNNs (Convolutional Neural Networks) because of its high efficiency, quick convergence, and gradient vanishing prevention. In our study, the input data are 3D MRI images with high dimensions. Accordingly, our diagnosis network is 3D CNN. It is necessary to use such an efficient and effective activation function. Then, we add a BN layer [34] after each convolutional layer to reduce data fluctuations and accelerate training speed. MaxPooling [35] is executed to increase receptive fields. In order to avoid overfitting, a dropout [36] is used in all fully connected layers. Finally a softmax layer is used for prediction. The specific network settings are shown in Table 1.

#### 3.3.2. The Training of the Diagnosis Network

We use $D_{k}\;=\;\left(w_{1}^{k},w_{2}^{k},\cdot \cdot \cdot ,w_{N}^{k}\right)$ to represent the weight vector of samples in the *k*th classifier, where *N* is the number of samples and $w_{i}^{k}\left(1\;\leq \;i\;\leq \;N\right)$ is the weight of the *i*th sample in the *k*th classifier. The sample weights in the first classifier are initialized as:

(3) $$D_{1}\;=\;\left(w_{1}^{1},\cdot \cdot \cdot ,w_{i}^{1},\cdot \cdot \cdot ,w_{N}^{1}\right),\;w_{i}^{1}\;=\;\frac{1}{N},\;i\;=\;1,2,\cdot \cdot \cdot ,N.$$

In order to adjust the weight of each sample in next classifier and achieve adaptive fusion, the error of each classifier is defined as follows:

(4) $$e_{k}\;=\;\sum_{i=1}^{N}w_{i}^{k}I\left(C_{k}\left(x_{i}\right),y_{i}\right),$$

where $e_{k}$ represents the error of the *k*th classifier, and $C_{k}\left(x_{i}\right)$ is the output corresponding to the sample $x_{i}$ in the *k*th classifier. $y_{i}$ represents the ground truth label of $x_{i}$, and I(·) is an indicator function, as shown in Equation (5).

(5) $$I\left(C_{k}\left(x_{i}\right),y_{i}\right)\;=\;\left\{\begin{array}{c} 1\;C_{k}\left(x_{i}\right)\;\neq \;y_{i}\\ 0\;C_{k}\left(x_{i}\right)\;=\;y_{i}. \end{array}\right.$$

By the error in Equation (4), we can learn an adaptive fusion weight for each classifier as follows:

(6) $$\alpha_{k}\;=\;\frac{1}{2}\log\frac{1\;-\;e_{k}}{e_{k}}$$

where $\alpha_{k}$ represents the fusion weight of the *k*th classifier. According to Equation (6), the classifier with a larger error will be assigned a smaller fusion weight and vice versa. According to the error $e_{k}$ of the *k*th classifier, the weight $w_{i}^{k+1}$ of each sample $x_{i}$ in the (k + 1)th classifier is adjusted as:

(7) $$w_{i}^{k+1}\;=\;\frac{w_{i}^{k}}{Z_{k}}\exp\left(-\alpha_{k}y_{i}C_{k}\left(x_{i}\right)\right),\;i\;=\;1,2,\cdot \cdot \cdot ,N$$

(8) $$Z_{k}\;=\;\sum_{i=1}^{N}w_{i}^{k}\exp\left(-\alpha_{k}y_{i}C_{k}\left(x_{i}\right)\right),$$

where $Z_{k}$ is the normalization factor of the *k*th classifier. In the *k*th classifier, if $x_{i}$ is classified incorrectly, that is, $C_{k}\left(x_{i}\right)$ and $y_{i}$ are inconsistent, its weight will increase in the (k + 1)th classifier.

#### 3.3.3. Loss Function

The cross-entropy loss is frequently used for image classification. However, the traditional cross-entropy loss does not consider the difference between samples. In this paper, we improve the traditional cross-entropy loss by incorporating the weight of each sample, as shown in Equation (9). From the training process of the diagnosis network, we can know that the weight of each sample may be adjusted in different classifiers. So each classifier has a separate loss function w.r.t sample weights.

(9) $$\begin{array}{c} loss^{k}\;=\;\frac{1}{N}\sum_{i=1}^{N}-w_{i}^{k}[y_{i}\;\cdot \;\log\left(C_{k}\left(x_{i}\right)\right)\\ \;+\;\left(1-y_{i}\right)\;\cdot \;\log\left(1-C_{k}\left(x_{i}\right)\right)] \end{array}$$

where $loss^{k}$ is the loss function of the *k*th classifier.

### 3.4. Fusion Diagnosis

In the training process of each classifier, its error is calculated independently by Equation (4), and the fusion weight of each classifier in diagnosis is determined by its error as shown in Equation (6). The commonly used ensemble learning prediction method is based on voting. Each classifier votes on the prediction result, and the prediction with the most votes is the final result. However, if some classifiers are not trained accurately, their prediction results are unreliable. Therefore, it is necessary to assign a higher weight to a classifier with less error in the fusion diagnosis. In this paper, we adaptively fuse the results of various classifiers according to their errors as follows:

(10) $$f\left(x\right)\;=\;\sum_{k=1}^{K}\alpha_{k}^{}C_{k}^{}\left(x\right),$$

where *f* is our fusion diagnosis function. *x* is a denoised testing sample, and *K* is the number of classifiers.

## 4. Experiments

### 4.1. Dataset

We conduct experiments on an ADNI dataset from Alzheimer’s Disease Neuroimaging Initiative [37]. The data are 3D MRI images. The imaging protocol parameters are shown in Table 2. The image format is neuroimaging informatics technology initiative (NIFTI).

**Figure 4:**
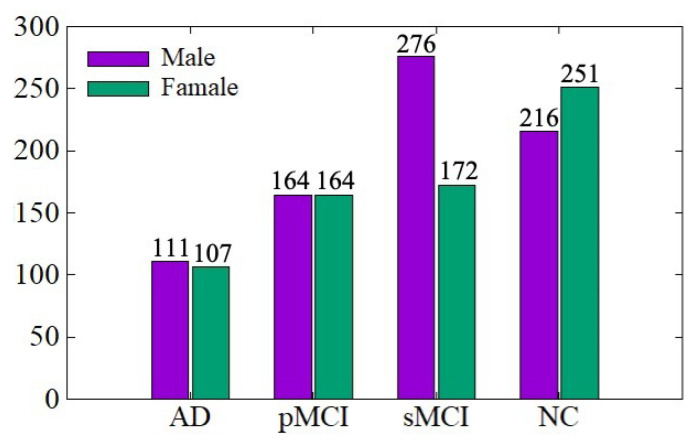
ADNI data distribution.

The ADNI data distribution is shown in Figure 4. There are a total of 1461 MRI images, including 767 males and 694 females, aged between 54 and 96 years. We conduct training and testing on the ADNI dataset to verify the effectiveness of the data denoising module and diagnosis network module proposed in this paper. The ratio of training set to the testing set is 7:3. In order to be consistent with the experimental results of existing work for comparison, we conduct AD vs. NC and sMCI vs. pMCI diagnostic experiments, in which the sMCI-image group and pMCI-image group adopt the same denoising method as AD-image group and NC-image group.

### 4.2. Experiment Setting

#### 4.2.1. Implement Details

The experiments are implemented on a computer with a single GPU (NVIDIA RTX 2080 Ti) and the platform of Pytorch. To accelerate the training process and avoid local minimums, we use the Adam algorithm [38] as the training optimizer. The hyperparameters of our network are as follows: the batchsize is set to 4, and the epoch is set to 50. The initial learning rate equals to $10^{-4}$, and the decay rate is $10^{-4}$.

#### 4.2.2. Performance Evaluation

In our diagnosis tasks, the predicted results are divided into the following four situations: True Positive (TP), True Negative (TN), False Positive (FP), and False Negative (FN). Among them, TP indicates that a positive sample is predicted to be a positive sample. TN indicates that a negative sample is predicted to be a negative sample. FP indicates that a negative sample is predicted to be a positive sample. FN indicates that a positive sample is predicted to be a negative sample. We employ the following four indicators to evaluate our diagnosis model: Accuracy, Specificity, Sensitivity, and Area under curve (AUC). Accuracy is the proportion of correctly diagnosed samples among the whole testing samples, as shown in Equation (11).

(11) $$Accuracy\;=\;\frac{TP\;+\;TN}{TP\;+\;TN\;+\;FP\;+\;FN}$$

Specificity represents the percentage of correctly diagnosed samples in disease-free testing samples, as shown in Equation (12).

(12) $$Specificity\;=\;\frac{TN}{TN\;+\;FP}$$

Sensitivity reflects the ability of a model to recognize Alzheimer’s patients in all positive samples, as shown in Equation (13).

(13) $$Sensitivity\;=\;\frac{TP}{TP\;+\;FN}$$

AUC is obtained by summing the area under a receiver operating characteristic (ROC) curve. The ROC curve expresses the sensitivity of a classifier to the distance between positive and negative samples, but the curve cannot clearly show which classifier is better. So, AUC is used as the numerical indicator for comparison. The larger the AUC, the more accurate the corresponding model.

### 4.3. Experimental Results

#### 4.3.1. Parameter Analysis

**Figure 5:**
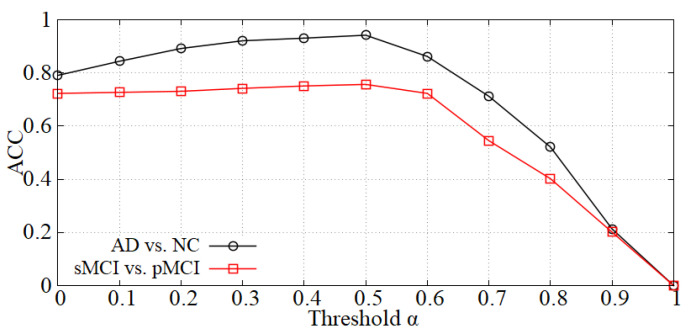
The effect of α on diagnostic accuracy.

Analysis of the threshold α in the data denoising module. As mentioned in Section 3.2, we first perform data denoising before inputting samples into the diagnosis network. The α value will influence the denoising effect, thereby affecting the diagnostic accuracy. The curve of accuracy versus α is shown in Figure 5 and the accuracy reaches a peak at α = 0.5 in each diagnostic task (i.e., AD vs. NC and sMCI vs. pMCI). When α is less than 0.5, the boundary noise of images is less cropped and the effect is not ideal. On the contrary, when α is larger than 0.5, some useful information may be cropped. Thereby, we set α = 0.5 in our experiments.

**Figure 6:**
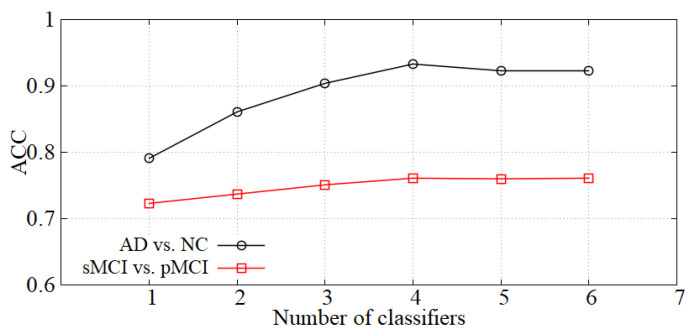
The effect of *K* on diagnostic accuracy.

Analysis of the number of classifiers. We incorporate the idea of ensemble learning in our diagnosis network and train multiple classifiers for fusion diagnosis. The diagnosis effect is improved by increasing the loss of hard samples and reducing the weights of the classifiers with high errors. We show the accuracy of our model with the number of classifiers *K* in Figure 6. When *K* is small, the diagnostic effect is improved with *K* increasing. When *K* is greater than 4, the performance is basically unchanged, so the number of classifiers *K* is set to 4 in our experiments.

#### 4.3.2. Effectiveness of Data Denoising Module

**Figure 7:**
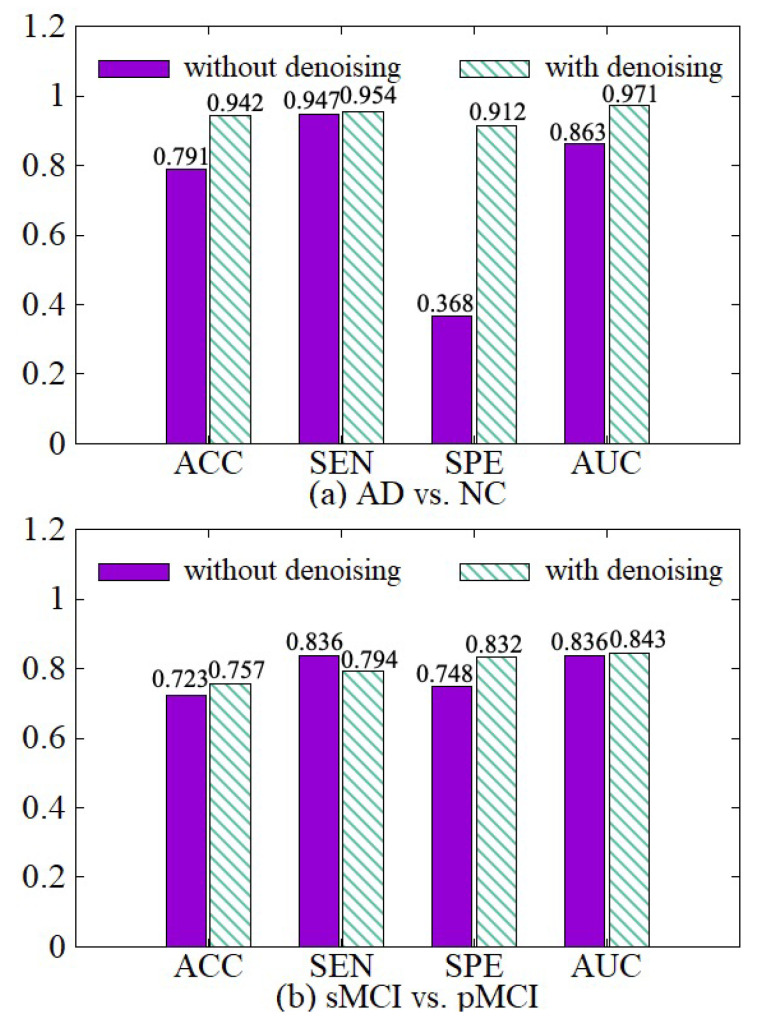
Effectiveness of the data denoising module. (**a**) shows the performance of AD vs. NC diagnostic task. (**b**) shows the performance of sMCI vs. pMCI diagnostic task.

In order to illustrate the effect of the data denoising module, we compare the model with the denoising module and the model without the denoising module in both AD vs. NC and sMCI vs. pMCI tasks. The experimental results are shown in Figure 7. The experimental results show that we can get better diagnostic effects by removing the useless information of 3D MRI boundaries. ACC, SPE, and AUC have been significantly improved in the two diagnostic tasks. In the process of extracting the features of the raw data, the network calculates a large amount of boundary noise which will disturb feature extraction and diagnosis. After we crop the boundary noise of images, the useless voxels are reduced, making the diagnosis effect better in two tasks. Moreover, the training speed is also improved significantly after data noise reduction, as shown in Table 3.

**Figure 8:**
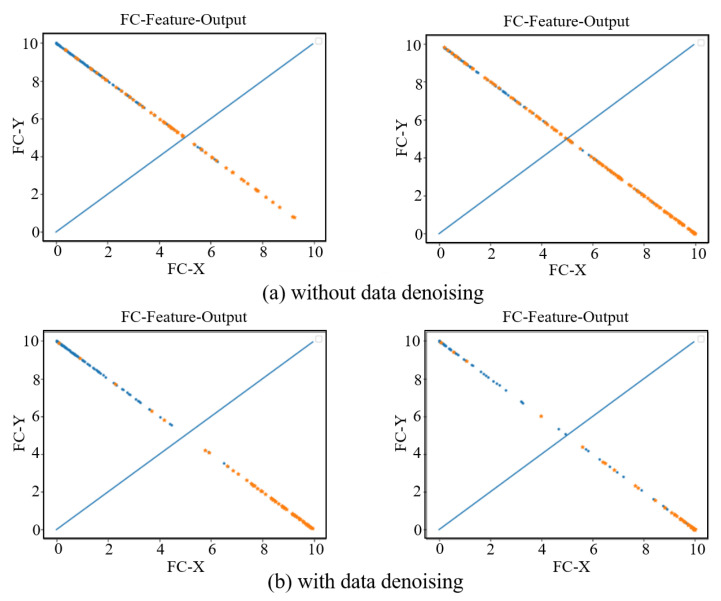
Fully connected layer feature visualization. The left two subfigures represent the AD vs. NC diagnosis task, and the right two subfigures represent the sMCI vs. pMCI diagnosis task. (**a**) shows the feature visualization without data denoising. (**b**) shows the feature visualization with data denoising.

In order to see the effect of the data denoising module on the AD diagnosis, we visually show the features extracted from the last fully connected layer in the AD vs. NC and sMCI vs. pMCI diagnosis tasks. As show in Figure 8, the left sub-figure represents the AD vs. NC diagnosis task, and the right sub-figure represents the sMCI vs. pMCI diagnosis task. The features of a sample can be represented by a point (FC-X, FC-Y) in a two-dimensional space, where (FC-X, FC-Y) is the feature vector from the last fully connected layer of our network. The softmax function is used after the fully connected layer, so the points corresponding to all samples are on the straight line x + y = 1. In Figure 8, a blue dot indicates that the label is a positive sample, and an orange dot indicates that the label is a negative sample. In the final prediction, if y > x, the test sample is predicted as a positive sample, otherwise it is a negative sample. Therefore, the dividing line of the network model can be regarded as the straight line y = x. From this, it can be seen that the data denoising module improves the network’s ability to distinguish between positive samples and negative samples.

#### 4.3.3. Effectiveness of Diagnosis Network Module

**Figure 9:**
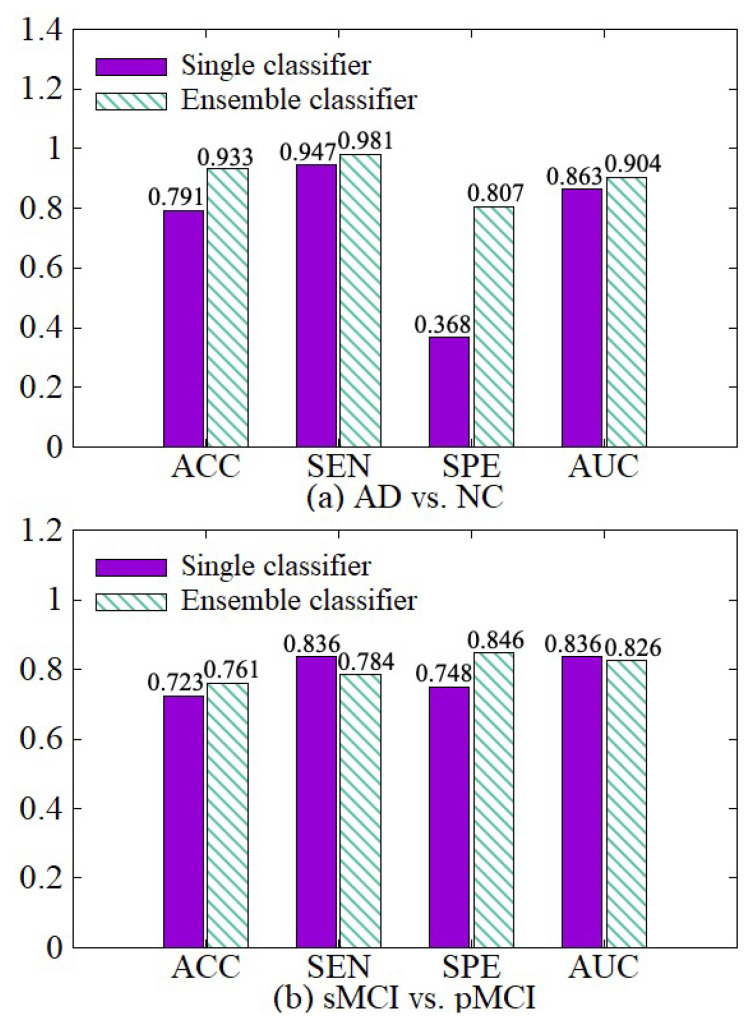
Effectiveness of the diagnosis network module. (**a**) shows the performance of AD vs. NC diagnostic task. (**b**) shows the performance of sMCI vs. pMCI diagnostic task.

We make a comparative experiment which uses a single classifier and our diagnosis network, which incorporates the idea of ensemble learning and contains multiple classifiers. The experimental results are shown in Figure 9. After the diagnosis network module is used, the weights of hard samples are increased, so that the network strengthens the learning of the hard samples. Besides, the diagnosis network learns the fusion weights of various classifiers and the diagnosis results of multiple classifiers are adaptively fused together with their weights in the final fusion diagnosis. So the results of our diagnosis network on the four indicators ACC, SEN, SPE, and AUC are better than the single classifier in both AD vs. NC diagnosis tasks.

#### 4.3.4. Effectiveness of the Improved Loss

**Figure 10:**
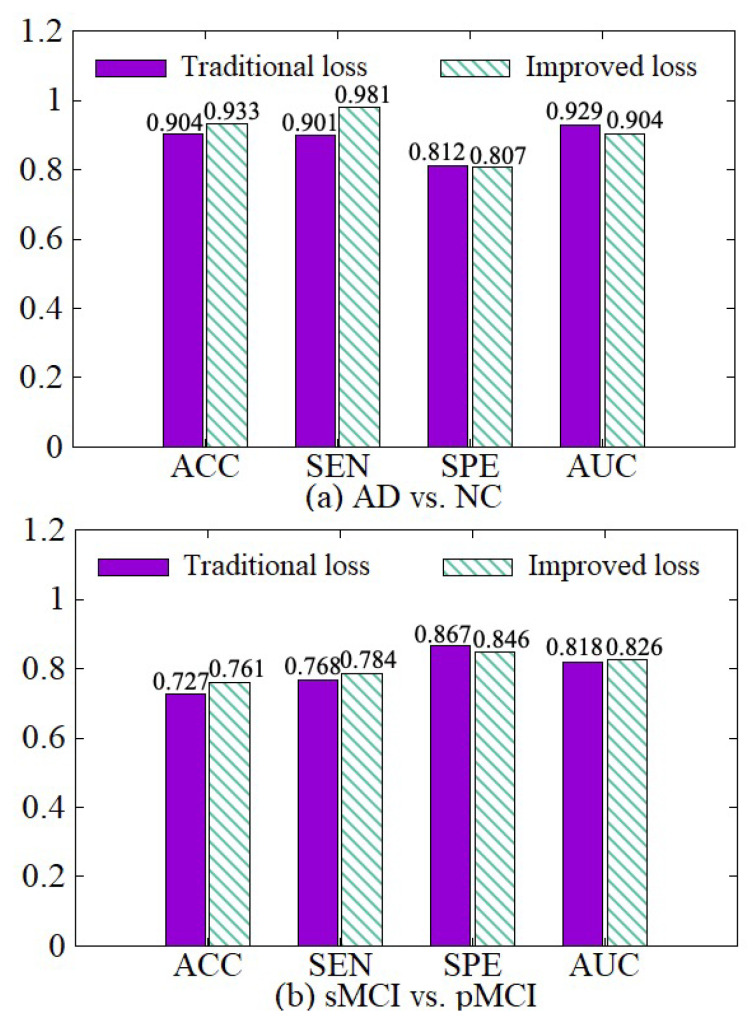
Effectiveness of loss modification. (**a**) shows the performance of AD vs. NC diagnostic task. (**b**) shows the performance of sMCI vs. pMCI diagnostic task.

We compare the diagnosis results based on the traditional cross-entropy loss and our improved loss, as shown in Figure 10. On the basis of the traditional cross-entropy loss, we incorporate the weights of various samples to increase the punishment for hard samples in the training process, so the network can focus on learning hard samples. Besides, the loss function of each classifier is adaptively adjusted with its error. Therefore, the diagnosis results based on our improved loss on the four indicators are better than the traditional cross-entropy loss.

#### 4.3.5. Effectiveness of the Model including Data Denoising and Diagnosis Network

**Figure 11:**
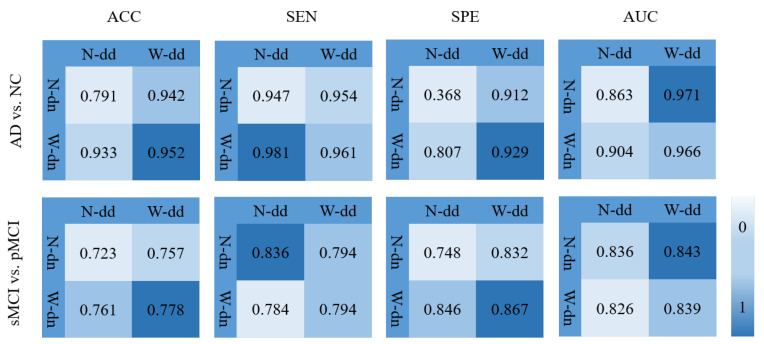
Effectiveness of the model including data denoising and diagnosis network.

In order to verify the effectiveness of the model including the data denoising and the diagnosis network, we design an experiment as shown in Figure 11 where various background colors are used to indicate different diagnosis effects, and a darker color indicates a better effect. In Figure 11, W-dd means that the data denoising module is used, N-dd means that the data denoising module is not used. W-dn means that the diagnosis network module is used, and N-dn means that the network is trained with a single classifier. From the experimental results, it can be seen that W-dd and W-dn have achieved good results in both classification tasks. Among them, the two indicators of ACC and SPE have better results. The not very obvious effect of the AUC may be caused by the imbalance of positive and negative samples. In conclusion, the two modules proposed in this paper are effective for the diagnosis of this disease.

#### 4.3.6. Comparisons with Other Methods

To verify the effectiveness of our work, we compare our method with other AD diagnosis methods. It is worth noting that all the baselines are using 3D CNN as the feature extraction basis neural network. In Table 4, a brief description of the state-of-the-art studies using ADNI structural MRI data AD vs. NC diagnostic task and sMCI vs. pMCI diagnostic task is presented. The experimental results show that our method has good performance on both two tasks. Our proposed method outperforms the previous work on both tasks, with an accuracy of 95.2% for AD vs. NC, 77.8% for sMCI vs. pMCI. This fully indicates that our method is effective for the diagnosis of Alzheimer’s disease.

**Figure 12:**
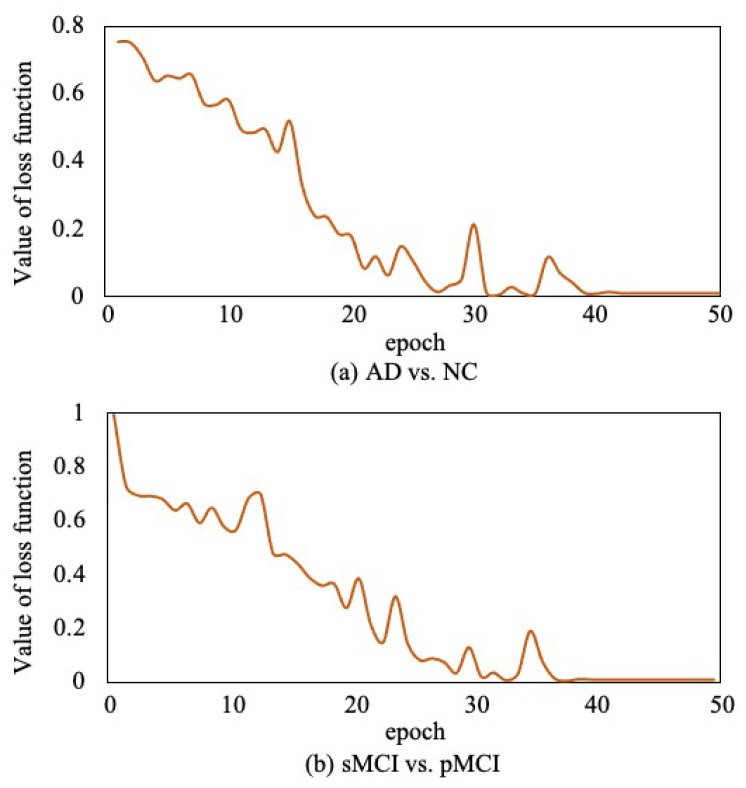
Convergence curves of loss function.

Moreover, to verify the convergence of the proposed method, as shown in Figure 12, we report the loss function value at each epoch in AD vs. NC diagnosis task and sMCI vs. pMCI diagnosis task. It can be observed that the objective function value decreases in the first 40 epochs and then gradually converges within 50 epochs.

## 5. Conclusions

In this study, a novel model with ensemble learning classifiers and 3D convolutional neural networks was proposed to diagnose Alzheimer’s disease. First, we present an effective data denoising method to reduce data noise so that we can improve the accuracy of diagnosis while speeding up the training process. Second, we propose a diagnosis network with multiple classifiers incorporating the ensemble learning idea to further improve the diagnosis effect, where the limited 3D MRI samples are repeatedly utilized in various classifiers and the weight of each sample in various classifiers can be learned to focus on hard samples. The corresponding loss function is also improved. Next the diagnostic results of various classifiers are adaptively fused based on their separate errors. Finally, extensive experiments have been done on the ADNI dataset to verify the effectiveness of our model. The experimental results indicate that the model proposed in this paper improves the training speed of the neural network and achieves 95.2% accuracy rate in AD vs. NC task and 77.8% accuracy rate in sMCI vs. pMCI task. Compared with several state-of-the-art computer-aided diagnostic methods, our proposed method has demonstrated better or at least comparable classification performance. In this paper, the threshold setting in the data denoising module is fixed for different images by experiments. In the future, we will consider employing dynamic thresholds for various images so as to minimize the loss of effective voxels while reducing noise and further improve diagnosis effect. Once again, thank you very much for your valuable comments and suggestions.

## Figures and Tables

**Table 1:** Basic setting of each classifier.

Layer	Operation	Size	Input	Output
0	conv(bn)	3 × 3 × 3 × 8	192 × 192 × 160 × 1	192 × 192 × 160 × 8
1	maxpooling	2 × 2 × 2	192 × 192 × 160 × 8	96 × 96 × 80 × 8
2	conv(bn)	3 × 3 × 3 × 16	96 × 96 × 80 × 8	96 × 96 × 80 × 16
3	maxpooling	2 × 2 × 2	96 × 96 × 80 × 16	48 × 48 × 40 × 16
4	conv(bn)	3 × 3 × 3 × 32	48 × 48 × 40 × 16	48 × 48 × 40 × 32
5	conv(bn)	3 × 3 × 3 × 32	48 × 48 × 40 × 32	48 × 48 × 40 × 32
6	maxpooling	2 × 2 × 2	48 × 48 × 40 × 32	24 × 24 × 20 × 32
7	conv(bn)	3 × 3 × 3 × 64	24 × 24 × 20 × 32	24 × 24 × 20 × 64
8	conv(bn)	3 × 3 × 3 × 64	24 × 24 × 20 × 64	24 × 24 × 20 × 64
9	maxpooling	2 × 2 × 2	24 × 24 × 20 × 64	12 × 12 × 10 × 64
10	conv(bn)	3 × 3 × 3 × 64	12 × 12 × 10 × 64	12 × 12 × 10 × 64
11	conv(bn)	3 × 3 × 3 × 64	12 × 12 × 10 × 64	12 × 12 × 10 × 64
12	maxpooling	2 × 2 × 2	12 × 12 × 10 × 64	6 × 6 × 5 × 64
13	fc	2048	6 × 6 × 5 × 64	2048
14	fc	2048	2048	2048
15	fc	2	2048	2

**Table 2:** Setting of the imaging protocol. The left is the imaging parameter, and the right is the corresponding value.

Protocol Parameter	Value
Acquisition Plane	Sagittal
Acquisition Type	3D
Field Strength	1.5 tesla
Slice Thickness	1.2 mm
TE	3.5–3.7 ms
TI	1000.0 ms
TR	3000.0 ms
Weighting	T1

**Table 3:** Comparison of training time with or without data denoising.

Training Time(s)	AD vs. NC	sMCI vs. pMCI
Without denoising	12,093	13,784
With denoising	6399	7273

**Table 4:** Comparisons with other methods.

Methods	AD vs. NC				sMCI vs. pMCI			
	ACC	SEN	SPE	AUC	ACC	SEN	SPE	AUC
Suk et al. [18]	0.92	0.92	0.95	0.97	0.72	0.37	0.91	0.73
Ortiz et al. [20]	0.90	-	-	0.95	-	-	-	-
Liu et al. [16]	0.91	0.88	0.94	0.96	0.77	0.42	0.82	0.78
Karasawa et al. [27]	0.94	-	-	-	-	-	-	-
Cui et al. [11]	0.92	0.91	0.94	0.97	0.75	0.73	0.76	0.80
Feng et al. [30]	0.95	0.98	0.93	0.97	-	-	-	-
Lian et al. [19]	0.90	0.82	0.97	0.95	0.81	0.53	0.85	0.78
Alinsaif et al. [2]	-	-	-	-	0.70	0.60	0.80	-
Ours	0.95	0.96	0.93	0.97	0.78	0.79	0.87	0.84

## Data Availability

The dataset used in this study was obtained from Alzheimer’s Disease Neuroimaging Initiative (ADNI). More information regarding ADNI can be obtained from the following link: http://adni.loni.usc.edu/ (accessed on 16 November 2021).
